# Editorial: Navigation and Perception for Autonomous Surface Vessels

**DOI:** 10.3389/frobt.2022.918464

**Published:** 2022-05-20

**Authors:** Fausto Ferreira, Alberto Quattrini Li, Ørnulf J. Rødseth

**Affiliations:** ^1^ Faculty of Electrical Engineering and Computing, University of Zagreb, Zagreb, Croatia; ^2^ Department of Computer Science, Dartmouth College, Hanover, NH, United States; ^3^ SINTEF Ocean, Trondheim, Norway

**Keywords:** autonomous surface vehicle (ASV), autonomous ship, navigation and control, perception, reinforcement learning, machine learning, collision avoidance (CA)

While autonomous navigation for ground robots has been studied for a several decades, a large gap is still present for autonomous vehicles in the marine domain. Challenges include unpredictable dynamics, mostly unstructured environments, and comparatively high cost in terms of logistics and vehicles to conduct experiments. Solving such challenges require new methods that improve the navigation and perception abilities of autonomous surface vessels. Thus, this special research topic in the journal “Frontiers in Robotics and AI” aims at covering new advances around these issues. Four articles were selected for publication and the result is this edition of the journal. In the following we will give a brief overview of the four papers.

Deep Reinforcement Learning (RL) techniques have been thoroughly used in the field of Autonomous Surface Vessels both for perception and for navigation. However, many works propose a DRL technique and compare with a limited number of state-of-the-art methods. “Comparing deep reinforcement learning algorithms’ ability to safely navigate challenging waters” (Larsen et al.) addresses a so-much needed comparison of different techniques. Specifically, the authors tackle the trade-off between collision avoidance and path planning and analyse the behaviour impact in this trade-off depending on the choice of RL technique. The role of the reward function in this multi-objective optimization is discussed as well.

To generalize obstacle avoidance from ground mobile robots to marine vehicles and to bridge the gap between simulation and reality, “Robust ASV Navigation Through Ground to Water Cross-Domain Deep Reinforcement Learning” (Lambert et al.) proposes a navigation method based on a deep reinforcement learning framework for high-level control, integrated with low-level controllers specific to the vehicle. The paper demonstrates the cross-domain generalizability of the proposed method by training a Deep Q Network for a simulated Autonomous Ground Vehicle (AGV) with navigation tasks. It then successfully implements and tests the proposed DRL method on a real AGV as well as an Autonomous Surface Vehicle (ASV) in different scenarios without any re-training. While the paper can be expanded to handle more complex scenarios, e.g., with dynamic obstacles, the paper provides a good methodology for training reinforcement-learning based approaches in domains that are comparatively easier and lower cost for deployments compared to challenging target domains, as the marine one.

Deep RL are not the only techniques that can be used for autonomous navigation. The complexity of the issue requires a deep study of the field. Indeed, collision avoidance and safe navigation in environments where other crewed or uncrewed ships sail, are perhaps the biggest challenges for safe operation of autonomous surface vessels. In particular, the coexistence with other crewed ships require that autonomous vessels obey the rights of way for the sea, i.e. the international collision regulations or COLREG as they are called. “Autonomous Collision Avoidance at Sea: A Survey” (Burmeister and Constapel) gives a comprehensive overview of relevant literature in this field and systemizes the approach taken by each reviewed paper in terms of what specific part of the problem they address, how they control the vessel, what sensors they use, and what algorithm or method they employ. This also includes a cross reference between the most relevant rules in COLREG and the papers that address them. In addition, the paper includes an analysis of how the performance of the different methods or algorithms have been validated. It is interesting to note that only around 10% of the 48 papers surveyed in detail refer to *in-situ* tests. The paper gives a very good overview of an important research area for autonomous surface vessels, but it also makes clear that much more research and validation are required.

Finally, good perception and navigation algorithms are sometimes not enough for guaranteeing a minimum performance of the situational awareness functionality. “Dynamic semantic world models and increased situational awareness for highly automated inland waterway transport” (Baelen et al.) addresses the need for good dynamic semantic world models for better situational awareness. Both internal and external world models are described. One important characteristic of this work is the ability to dynamically adapt to new situations and the simplicity of interpretation in those dynamic situations by all actors involved. The contribution to explainability and performance in dynamic environments is a significant step towards safer navigation.

The editors wish to thank all the authors, reviewers, and the whole editorial team for the hard work and perseverance in making this Research Topic come through.

